# Biocontrol Potential and Functional Characteristics of *Bacillus sonorensis* A-5 Against Watermelon Fusarium Wilt

**DOI:** 10.3390/jof12040257

**Published:** 2026-04-02

**Authors:** Jian-Wei Jiang, Yue Qiu, Liu-Tong Ye, Jing-Xue Luo, Qianwen Nie, Yi Zhou

**Affiliations:** College of Agriculture, Yangtze University, Jingzhou 434025, China; 1329708325@163.com (Y.Q.); 18876079555@163.com (L.-T.Y.); l690769@163.com (J.-X.L.); nieqianwen95@126.com (Q.N.)

**Keywords:** biological control, *Fusarium oxysporum* f. sp. *niveum*, *Fon*, antagonistic mechanism

## Abstract

Fusarium wilt, caused by *Fusarium oxysporum* f. sp. *niveum* (*Fon*), severely restricts the sustainable development of the global watermelon industry. While conventional chemical fungicides of this disease have triggered prominent ecological issues, *Bacillus*-based microbial biocontrol, which combines inherent environmental compatibility with stable control efficacy, has emerged as a key green alternative to chemical management. However, the biocontrol potential of *Bacillus sonorensis* against this disease has not yet been fully investigated. In this study, we isolated 56 bacterial strains from healthy watermelon rhizosphere soil, and obtained a *Fon*-antagonistic strain A-5 with the strongest activity (70.15% mycelial inhibition rate), which was identified as *B. sonorensis* via polyphasic taxonomic analysis. In vitro assays showed that the sterile fermentation filtrate of strain A-5 had a maximum 81.05% inhibition rate against *Fon*, and its volatile organic compounds also significantly suppressed *Fon* growth, with broad-spectrum antifungal activity against four common phytopathogenic fungi. Functional tests confirmed that strain A-5 could secrete cell wall-degrading enzymes, produce siderophores and synthesize indole-3-acetic acid, and 17 antimicrobial secondary metabolite biosynthetic gene clusters were identified in its genome. Pot experiments verified that strain A-5 had a 78.04% relative control efficacy against watermelon Fusarium wilt, which significantly reduced seedling disease incidence and upregulated defense-related antioxidant enzyme activities in watermelon leaves. In general, *B. sonorensis* A-5 is a promising novel biocontrol agent for green management of watermelon Fusarium wilt.

## 1. Introduction

Watermelon (*Citrullus lanatus*) is a globally cultivated high-value horticultural crop, with its sustainable production continuously challenged by soil-borne Fusarium wilt [1]. This destructive disease is caused by the host-specific pathogen *Fusarium oxysporum* f. sp. *niveum* (*Fon*), which forms persistent chlamydospores that can survive in infested soil for more than a decade, leading to severe continuous cropping obstacles and yield losses of up to 80% in heavily affected fields [2]. To date, effective management of this disease remains a major challenge in agricultural production. Traditional strategies, including resistant variety breeding and crop rotation, are limited by the narrow resistance spectrum of available varieties and long implementation cycles [2]. Chemical fungicide application, particularly triazole agents and carbendazim seed treatment, is still the most widely used method for Fusarium wilt control [3]. However, long-term overuse of chemical fungicides has driven the emergence of fungicide-resistant *Fon* populations, along with a series of intractable issues including pesticide residues in agricultural products, soil micro-ecological imbalance, and biodiversity loss in farmland ecosystems [4,5,6,7,8].

Biological control using beneficial microorganisms and their metabolites offer a safe, eco-friendly alternative to chemical fungicides for Fusarium wilt management [9,10,11,12]. Biocontrol bacteria suppress pathogen infection through multiple complementary mechanisms, including the secretion of antimicrobial metabolites and lytic enzymes, induction of plant systemic resistance, and nutritional competition with pathogens in the rhizosphere [13,14]. Unlike chemical pesticides that easily cause residual pollution and soil microecological imbalance, antagonistic bacteria have significantly lower environmental risks, and most of them can adapt to the rhizosphere environment and maintain the stability of soil microbial communities, making them a promising eco-friendly control strategy for watermelon Fusarium wilt [15,16]. Previous studies have identified multiple microbial taxa with biocontrol potential against *Fon*, including *Bacillus amyloliquefaciens* [16], *Klebsiella aerogenes* [17], *Streptomyces alfalfa* [18] and *Paenibacillus polymyxa* [19]. However, most of these candidate strains are still limited to laboratory research due to unstable control efficacy in field conditions, creating an urgent need to screen novel, efficient, and environment-stable biocontrol strains for watermelon Fusarium wilt control.

*Bacillus* species are among the most promising candidates for commercial biocontrol agent development, due to their universal distribution in natural environments, strong environmental adaptability, and well-documented safety in agricultural applications. These bacteria can form stress-resistant endospores under adverse conditions, tolerating high temperature, drought, and UV radiation, which gives them significant advantages in formulation development and field application [18]. Meanwhile, *Bacillus* strains employ diverse biocontrol and plant growth-promoting mechanisms: they secrete cell wall-degrading enzymes (proteases, cellulases, glucanases) to destroy pathogen cellular structure, produce lipopeptide and polypeptide antibiotics to inhibit pathogen growth, synthesize phytohormones (such as indole-3-acetic acid, IAA) to promote plant root development, and secrete high-affinity siderophores to limit iron availability for pathogens via nutritional competition [20,21]. While the biocontrol potential of *B. subtilis*, *B. amyloliquefaciens*, and *B. velezensis* against watermelon Fusarium wilt has been extensively validated [22,23,24], the biocontrol efficacy, functional characteristics, and action mechanisms of *Bacillus sonorensis* against this disease remain largely uncharacterized, with no systematic reports on its application in watermelon Fusarium wilt management.

Therefore, the main objective of this study was to screen efficient antagonistic bacteria against *Fon* from watermelon rhizosphere, and systematically evaluate the biocontrol potential and functional characteristics of the target strain. Here, we isolated rhizosphere bacteria from healthy watermelon plants, and obtained a high-efficiency *Fon*-antagonistic strain A-5 identified as *Bacillus sonorensis* via polyphasic taxonomic analysis. We systematically characterized its antifungal spectrum, biocontrol-related functional traits and verified its control efficacy against watermelon Fusarium wilt. Whole-genome sequencing was further performed to reveal the genetic basis of its antimicrobial and plant growth-promoting properties. This study enriches the antagonistic bacterial resource pool for watermelon Fusarium wilt management, and provides a theoretical foundation for the development of novel biocontrol agents.

## 2. Materials and Methods

### 2.1. Isolation and Screening of Rhizosphere Antagonistic Bacteria

Rhizosphere soil samples of healthy watermelon plants were collected from 3 sampling plots in Taihu Farm, Jingzhou, Hubei Province (30.36′ N, 112.07′ E). For each sampling plot, we collected 9 independent soil samples from the 10–20 cm soil layer, which covers the main distribution zone of watermelon fibrous roots. Fresh rhizosphere soil tightly attached to the watermelon root surface was collected using a sterile shovel, and non-rhizosphere soil was removed by gentle shaking. All soil samples were transported back to the laboratory in an ice box, and processed for bacterial isolation. 10 g of thoroughly mixed soil was suspended in 50 mL sterile water containing glass beads, and shaken at 180 rpm for 25 min at 28 °C. The suspension was gradient diluted to 10^−6^, 10^−7^ and 10^−8^ in a laminar flow cabinet, then 100 μL of each dilution was spread on Luria-Bertani (LB) agar plates (5 g/L NaCl, 10 g/L tryptone, 5 g/L yeast extract, 20 g/L agar, pH 7.0). Plates were incubated at 28 °C for 2–3 d. Single colonies with distinct morphological characteristics were purified by repeated streaking, and preserved in 15% (*v*/*v*) glycerol at −80 °C for subsequent experiments.

The dual-culture assay was performed to screen antagonistic strains against *Fusarium oxysporum* f. sp. *niveum* (*Fon*, the causal agent of watermelon Fusarium wilt) on potato dextrose agar (PDA) plates (200 g/L potato infusion, 20 g/L glucose, 20 g/L agar, pH 7.0). The *Fon* strain was a single-spore purified laboratory-preserved strain, originally isolated from diseased watermelon plants in Jingzhou, Hubei Province, China, in 2020, and is preserved in the Laboratory of Plant Pathology, Yangtze University. A 6-mm mycelial plug of *Fon* was inoculated at the center of a PDA plate, and two parallel streaks of the candidate bacterial suspension (OD_600_ = 0.8, 2 mm width × 20 mm length) were inoculated 25 mm away from the fungal plug, with sterile water as the negative control. All plates were incubated in the dark at 28 °C until the mycelium in the control plate fully covered the plate. The colony radius of *Fon* was measured by the cross method, and the relative inhibition rate was calculated as: [(Rc − Rt)/Rc] × 100, where Rc is radial growth in the control, and Rt is radial growth in the treatment [25].

### 2.2. Polyphasic Taxonomic Identification of the Target Strain

#### 2.2.1. Morphological Observation

The target strain was streaked on LB agar plates and incubated at 28 °C for 48 h to observe colony morphological characteristics. The vegetative cell morphology was observed via scanning electron microscopy (SEM).

#### 2.2.2. 16S rDNA Sequencing and Phylogenetic Analysis

The target strain was cultured in LB liquid medium at 28 °C for 24 h, and total genomic DNA was extracted using a Bacterial Genomic DNA Extraction Kit (Vazyme, Nanjing, China). The 16S rDNA gene was amplified by PCR using universal primers 27F (5′-AGAGTTTGATCCTGGCTCAG-3′) and 1492R (5′-GGTTACCTTGTTACGACTT-3′), with the thermal cycling program referred to Jiang, et al. [26]. PCR products were sequenced by Tsingke Biotechnology Co., Ltd. (Wuhan, China). The corrected sequences were submitted to the NCBI GenBank database (https://www.ncbi.nlm.nih.gov/genbank/, accessed on 15 March 2025), and BLAST alignment was performed using the NCBI online BLAST tool (https://blast.ncbi.nlm.nih.gov/Blast.cgi, accessed on 15 March 2025). A phylogenetic tree was constructed using the neighbor-joining (NJ) method in MEGA 7.0 software, with bootstrap values set to 1000 replicates [27].

#### 2.2.3. Whole-Genome Sequencing and Taxonomic Validation

Whole-genome sequencing of the target strain was performed on the Illumina MiSeq platform by Smartgenomics Technology Co., Ltd. (Qingdao, China). Raw reads were quality-trimmed using fastp v0.20.0 [28], and de novo genome assembly was conducted with SPAdes v4.2.0 [29]. The average nucleotide identity (ANI) was calculated using FastANI 1.3 [30], and digital DNA-DNA hybridization (dDDH) values were computed via the Genome-to-Genome Distance Calculator (GGDC) 3.0 web server (https://ggdc.dsmz.de/ggdc.php, accessed 10 December 2025).

### 2.3. Genome Functional Annotation and Secondary Metabolite Prediction

Gene prediction of the assembled genome was performed using Prokka 1.14.6 with default parameters [31]. Functional annotation of predicted genes was conducted via BLAST alignment against the Clusters of Orthologous Groups (COG) database (2020 update, https://www.ncbi.nlm.nih.gov/research/cog/, accessed on 10 December 2025), Gene Ontology (GO) database (http://geneontology.org/, accessed on 10 December 2025), and Kyoto Encyclopedia of Genes and Genomes (KEGG) databases (http://geneontology.org/, accessed on 10 December 2025). Single-copy orthologous genes across *Bacillus* spp. genomes were identified using OrthoFinder 2.5.5 [32], and a genome-scale phylogenetic tree was constructed using the maximum likelihood (ML) method via RAxML-NG v. 0.9.0 [33]. Biosynthetic gene clusters (BGCs) of secondary metabolites were predicted using the antiSMASH 8.0 web server [34].

### 2.4. In Vitro Antifungal Activity Assays

#### 2.4.1. Antifungal Activity of Sterile Fermentation Filtrate

The target strain was inoculated into LB liquid medium and cultured at 180 rpm, 28 °C for 72 h. The culture was centrifuged at 12,000 rcf for 20 min at room temperature, and the supernatant was filtered through a 0.22 μm sterile filter membrane twice to obtain sterile fermentation filtrate. The filtrate was mixed with molten PDA medium at volume ratios of 5%, 10%, 20% and 40% (*v*/*v*) to prepare assay plates, with an equal volume of sterile LB medium added to the control group. A 6-mm *Fon* mycelial plug was inoculated at the center of each plate, and incubated at 28 °C for 5 d. The colony diameter of *Fon* was measured, and the relative inhibition rate was calculated according to Section 2.1.

#### 2.4.2. Antifungal Activity of Volatile Organic Compounds (VOCs)

The plate-in-plate method was used to evaluate the antifungal activity of VOCs produced by the target strain [35]. A 6-mm *Fon* mycelial plug was inoculated at the center of a 9-cm PDA plate (top layer). 100 μL and 200 μL of the bacterial suspension (OD_600_ = 0.8) were spread on 9-cm LB agar plates (bottom layer), respectively, with an equal volume of sterile LB medium as the control. The bottom and top plates were stacked, sealed with Parafilm, and incubated upside down at 28 °C for 3 d. The colony diameter of *Fon* was measured to calculate the relative inhibition rate of VOCs.

#### 2.4.3. Broad-Spectrum Antifungal Activity Assay

The dual-culture assay was used to determine the antifungal spectrum of the target strain against 4 common phytopathogenic fungi: *Phytophthora citrophthora* (citrus brown rot), *Verticillium dahliae* (cotton Verticillium wilt), *Fusarium fujikuroi* (rice bakanae disease), and *Colletotrichum camelliae* (tea anthracnose). The assay procedure was consistent with Section 2.1, and the relative inhibition rate against each pathogen was calculated according to Section 2.1.

### 2.5. Detection of Biocontrol and Plant Growth-Promoting Traits

#### 2.5.1. Extracellular Enzyme and Siderophore Production Assay

Two microliters of the bacterial suspension (OD_600_ = 0.8) were added to a 6-mm sterile filter paper disc, which was placed at the center of skim milk agar medium (protease detection) [36], β-1,3-glucanase detection medium [37], and Chrome Azurol S (CAS) medium (siderophore detection) [38], respectively. Plates were incubated at 28 °C for 48 h, and the production of corresponding enzymes or siderophores was determined by the presence of clear or orange-yellow degradation zones around the discs.

#### 2.5.2. Indole-3-Acetic Acid (IAA) Production Assay

The target strain was inoculated into LB liquid medium supplemented with 0.2 g/L L-tryptophan, and cultured at 180 rpm, 28 °C for 48 h. The culture was centrifuged at 10,000 rcf for 10 min, then 2 mL of the supernatant was mixed with 4 mL of Salkowski’s reagent and 2 drops of orthophosphoric acid, followed by incubation in the dark at room temperature for 30 min. The appearance of a pink color indicated positive IAA production [39].

### 2.6. Pot Experiment for Biocontrol Efficacy Evaluation

*Fon* was inoculated into 1.5 L potato dextrose broth (PDB) medium, and cultured at 180 rpm, 28 °C for 5 d. The culture was filtered through 3 layers of sterile gauze, and centrifuged at 4000 rcf for 10 min at room temperature. The spore pellet was resuspended in sterile PDB, adjusted to a concentration of 1 × 10^6^ spores/mL, and incubated at 28 °C for 60 min to prepare the final spore suspension. The nutrient soil was mixed with the spore suspension at a ratio of 2:1 (v/m) to prepare *Fon*-infested soil, with a final concentration of 2 × 10^5^ spores per gram of soil (Table S1). Germinated watermelon seeds (variety: Jingxin No. 1) were sown in seedling trays, and uniform seedlings at the two-true-leaf stage were transplanted into plastic pots (12 cm × 10 cm, 1 seedling per pot, with 10 independent biological replicates (10 seedlings) set for each treatment) filled with 500 g of *Fon*-infested soil. Two treatments were set in the experiment: (1) Mock group: irrigated with 20 mL sterile water per plant; (2) A-5 treatment group: irrigated with 20 mL of A-5 bacterial suspension (OD_600_ = 0.8) per plant. At 5 d after transplanting, the corresponding solution was irrigated into the root zone of each seedling, with applications repeated every 5 d for a total of 3 times. All seedlings were randomly placed in a greenhouse at 28 °C/25 °C (day/night), 16 h/8 h light/dark cycle, and 60% relative humidity. Disease symptoms were investigated at 10 d after the final irrigation, and disease severity was graded on a 0–4 scale: Grade 0, no visible disease symptoms on the whole plant; Grade 1, mild wilting with <25% of leaf area affected; Grade 2, wilting and leaf dehydration with 25–50% of leaf area affected; Grade 3, severe wilting and dehydration with >50% of leaf area affected; Grade 4, complete plant death. The disease index and relative control efficacy were calculated according to the formula described by Jiang, et al. [26]. Meanwhile, the activities of superoxide dismutase (SOD), peroxidase (POD), catalase (CAT), and malondialdehyde (MDA) content in watermelon leaves were determined using corresponding commercial assay kits (Solarbio, Beijing, China) according to the manufacturer’s instructions.

### 2.7. Data Analysis

All data are expressed as the mean ± standard error (SE). The Shapiro-Wilk test and Levene’s test were used to verify the normality and homogeneity of variances of all data, with significance set at *p* < 0.05. All analyses were performed in GraphPad Prism 9.1. One-way ANOVA with Fisher’s LSD post hoc test was used for parametric analysis of normally distributed continuous data. For disease index data, non-parametric tests were used. The Mann-Whitney U was used test for two-group comparisons.

## 3. Results

### 3.1. Isolation and Screening of Antagonistic Bacteria

**Figure 1:**
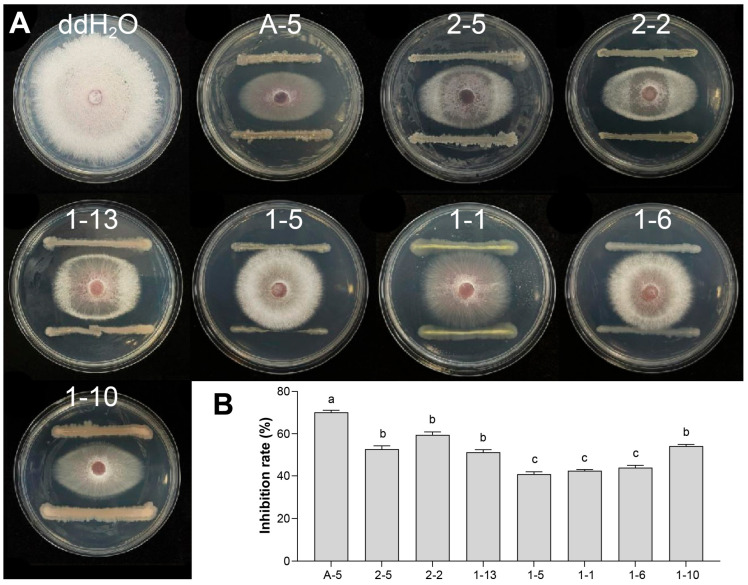
Antagonistic activity of candidate bacterial strains against *Fusarium oxysporum* f. sp. *niveum* (*Fon*). (**A**) Representative dual-culture assay plates of eight candidate strains against *Fon*, with sterile water (ddH_2_O) as the negative control. (**B**) Corresponding mycelial growth inhibition rates of the eight candidate strains against *Fon*. Different letters above bars indicate significant differences (*p* < 0.05).

Fifty-six bacterial strains were isolated from healthy watermelon rhizosphere soil using the dilution spread plate method. Their antagonistic activity against *Fon*, the causal agent of watermelon wilt, was assessed via the dual-culture assay. Eight strains exhibited significant inhibitory effects on the mycelial growth of *Fon*, with inhibition rates ranging from 40.77% to 70.15%. Among these, five strains showed inhibition rates exceeding 50%. Strain A-5 displayed the strongest antagonistic activity, with an inhibition rate of 70.15 ± 0.94% (Figure 1, Table S2). Therefore, strain A-5 was selected for further characterization and subsequent experiments.

### 3.2. Polyphasic Taxonomic Identification of Strain A-5

**Figure 2:**
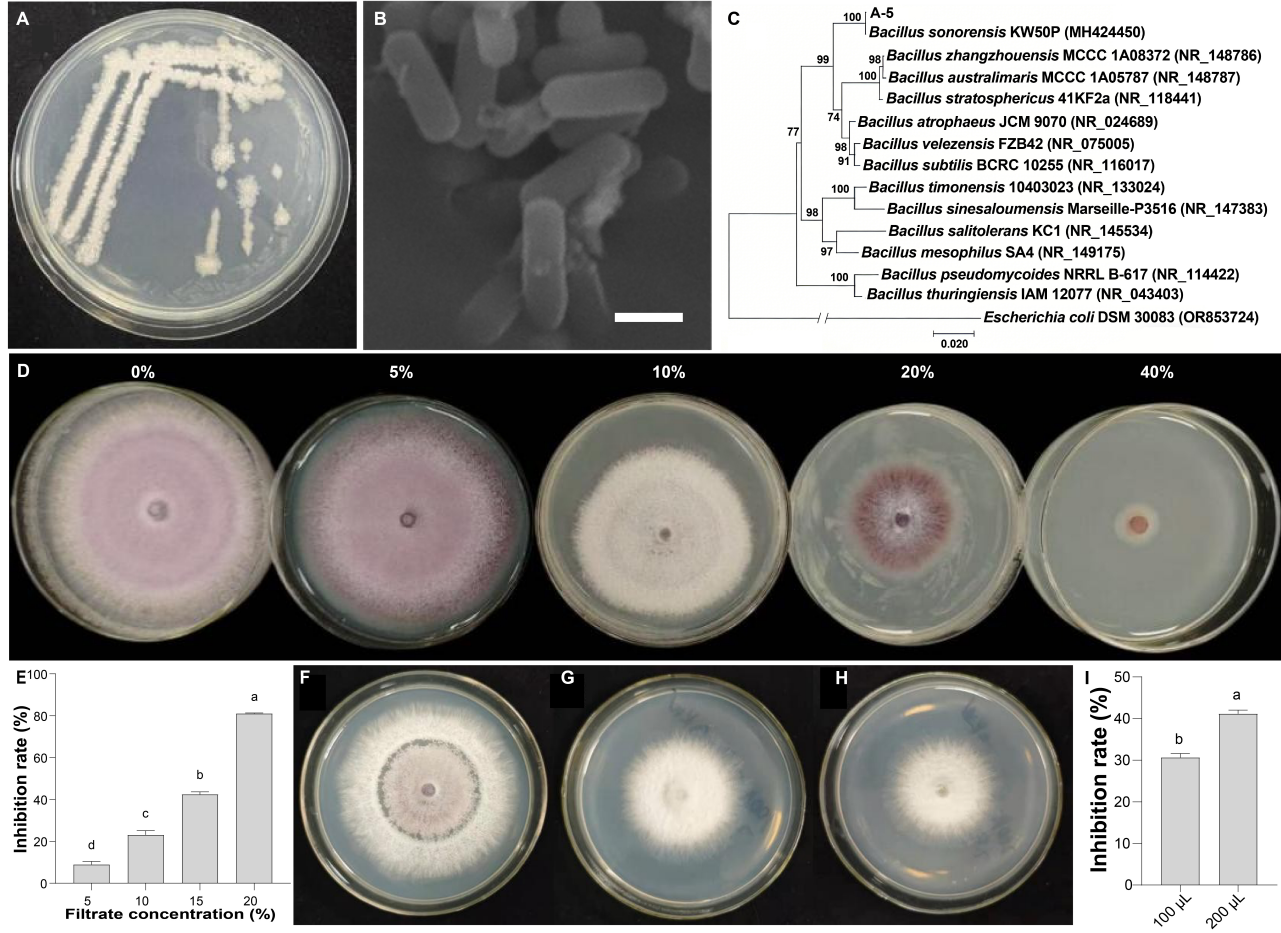
Identification and in vitro antifungal activity of strain A-5. (**A**) Colony morphological characteristics of strain A-5 cultured on LB agar for 48 h. (**B**) Scanning electron microscopy (SEM) image of strain A-5 vegetative cells (scale bar: 1 μm). (**C**) 16S rDNA-based neighbor-joining phylogenetic tree of strain A-5, with *Escherichia coli* DSM 30083 as the outgroup; bootstrap values were calculated from 1000 replicates. (**D**,**E**) Inhibitory effect of sterile fermentation filtrate of strain A-5 at different concentrations (0%, 5%, 10%, 20%, 40%) on *Fon* mycelial growth. (**F**–**I**) Inhibitory effect of VOCs on *Fon* mycelial growth: inoculated with sterile water (**F**), 100 (**G**) and 200 μL (**H**) of strain A-5 bacterial suspension, respectively. (**I**) Inhibition rates of VOCs against *Fon*. (**E**,**I**) Different letters above bars indicate significant differences (*p* < 0.05).

**Figure 3:**
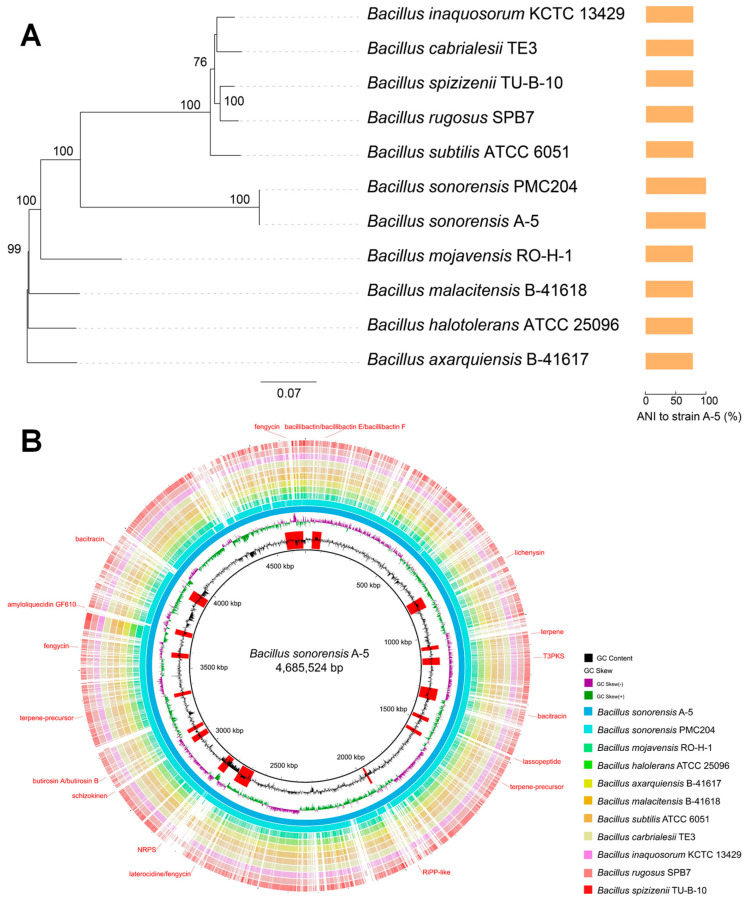
Genomic analysis of strain A-5. (**A**) Maximum likelihood (ML) phylogenetic tree based on single-copy orthologous gene sequences of *Bacillus* spp. genomes, with bar plots on the right showing the average nucleotide identity (ANI, %) of closely related strains relative to strain A-5. (**B**) Comparative genomic analysis between *B. sonorensis* A-5 and 10 *Bacillus* type strains.

Strain A-5 formed milky-white, mostly circular or irregular colonies on LB agar after 48 h of incubation. The colony surface was rough and wrinkled, with distinct folds at the center and margins, presenting a coarse, non-glossy texture (Figure 2A). SEM observation showed that the vegetative cells of strain A-5 were short rod-shaped, which was consistent with the typical morphological characteristics of the *Bacillus* genus (Figure 2B). Phylogenetic analysis based on 16S rDNA sequences showed that strain A-5 clustered tightly with *Bacillus sonorensis* (accession no. MH424450), forming an independent monophyletic clade with 100% bootstrap support (Figure 2C). Whole-genome sequencing further validated the taxonomic status of strain A-5 (genome accession no. JBVUNY000000000). The ANI value between strain A-5 and the type strain *B. sonorensis* PMC204 was 99.45%, and the dDDH value was 91%, both well above the prokaryotic species demarcation thresholds (ANI ≥ 95–96%, dDDH ≥ 70%). In contrast, ANI and dDDH values between strain A-5 and other closely related *Bacillus* species were all below the thresholds (Figure 3A, Table S3). These results firmly identified strain A-5 as a member of *B. sonorensis*.

### 3.3. In Vitro Antifungal Activity of Strain A-5 Against Fon

#### 3.3.1. Antifungal Activity of Sterile Fermentation Filtrate

The inhibitory effect of sterile filtrate from *B. sonorensis* A-5 on Fon was evaluated in vitro. The sterile filtrate of strain A-5 exhibited concentration-dependent inhibitory activity against mycelial growth. At a concentration of 5%, the inhibition rate was 8.92 ± 1.47%, with only a slight effect on fungal growth. The inhibition rate increased to 23.11 ± 1.96% at 10%, 42.44 ± 1.18% at 20%, and reached a maximum of 81.05 ± 0.29% at 40%, where the inhibitory effect was pronounced (Figure 2D,E, Table S4).

#### 3.3.2. Antifungal Activity of VOCs

The VOCs produced by strain A-5 also significantly inhibited the mycelial growth of *Fon*. When the control group mycelium covered more than two-thirds of the plate, the colony diameter of *Fon* in the treatment groups was significantly smaller. The inhibition rate was 30.63% for the 100 μL bacterial suspension treatment, and 41.16% for the 200 μL treatment, showing a dose-dependent effect (Figure 2F–I, Table S5).

#### 3.3.3. Broad-Spectrum Antifungal Activity

**Figure 4:**
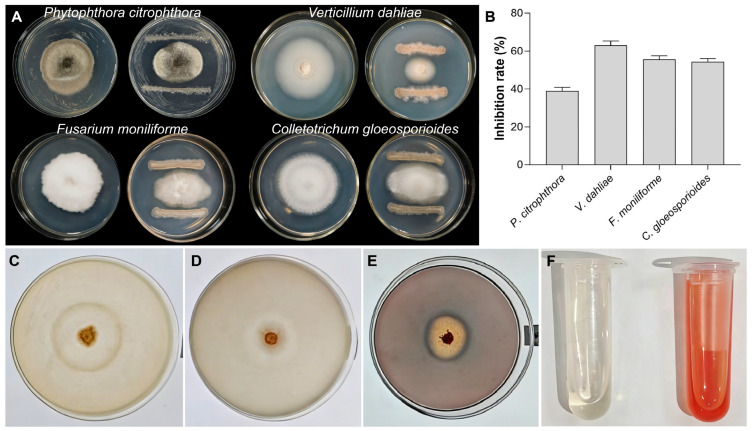
The antimicrobial spectrum of and biocontrol/plant growth-promoting traits of strain A-5. (**A**,**B**) Broad-spectrum antifungal activity of strain A-5 against four phytopathogenic fungi: *Phytophthora citrophthora*, *Verticillium dahliae*, *Fusarium fujikuroi*, and *Colletotrichum camelliae*. (**C**–**F**) Detection of functional traits of strain A-5: (**C**) β-1,3-glucanase production; (**D**) protease production; (**E**) siderophore production; (**F**) indole-3-acetic acid (IAA) production (left: negative control; right: positive pink reaction).

The antimicrobial spectrum of *B. sonorensis* A-5 was determined using the plate confrontation method against four pathogenic fungi. Strain A-5 exhibited broad-spectrum antifungal activity, with inhibition rates ranging from 33.47% to 60.88% (Figure 4A,B, Table S6). The strongest inhibitory effect was observed against *V. dahliae*, with an inhibition rate of 63.02 ± 2.34%. Inhibition rates exceeded 50% against three pathogens: *V. dahliae* (63.02 ± 2.34%), *F. moniliforme* (55.53 ± 1.93%) and *C. gloeosporioides* (54.26 ± 1.73%). In contrast, the inhibitory activity against *P. citrophthora* was relatively lower, at 38.92 ± 2.01%.

### 3.4. Biocontrol and Plant Growth-Promoting Traits of Strain A-5

Strain A-5 showed multiple biocontrol and plant growth-promoting functional traits. Distinct clear zones formed around the strain colonies on protease and β-1,3-glucanase detection media, confirming its ability to secrete these two cell wall-degrading enzymes (Figure 4C,D). A prominent orange-yellow degradation zone appeared around the colony on CAS medium, indicating positive siderophore production (Figure 4E). In the IAA production assay, the culture supernatant of strain A-5 turned pink after reacting with Salkowski’s reagent, verifying its capacity to synthesize IAA (Figure 4F).

### 3.5. Genome Assembly and Functional Analysis of Strain A-5

**Figure 5:**
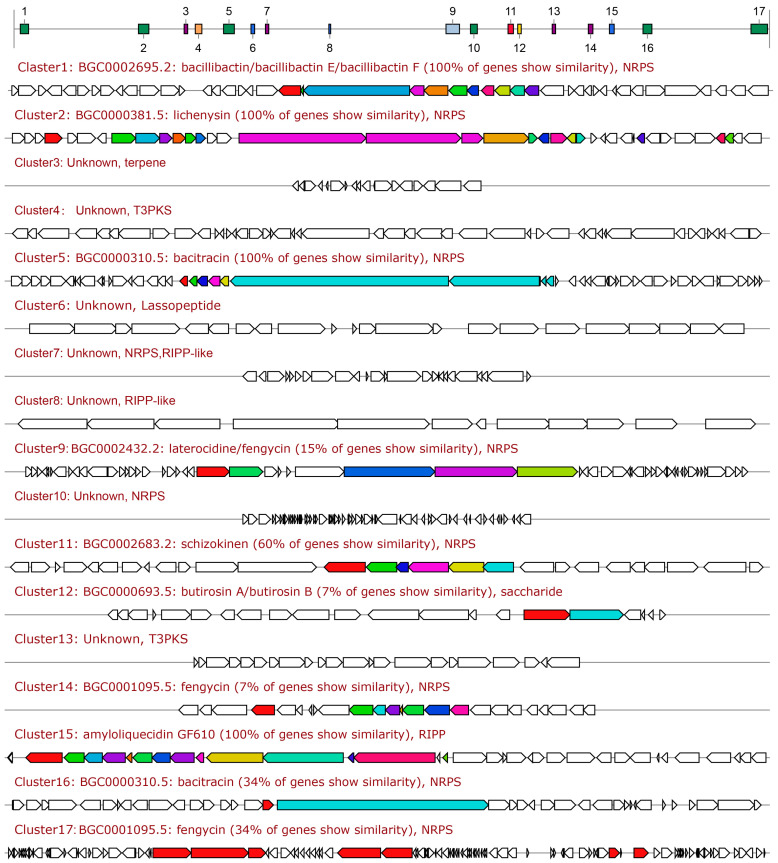
Prediction of secondary metabolite biosynthesis gene clusters (bgcs) in *Bacillus sonorensis* A-5 genome. A total of 17 BGCs were identified via antiSMASH 8.0 analysis, including non-ribosomal peptide synthetases (NRPS), type III polyketide synthases (T3PKS), ribosomally synthesized and post-translationally modified peptides (RiPPs), and terpene clusters. The similarity of core genes to known functional clusters in the MiBIG database is marked for each BGCs.

The complete genome of strain A-5 was 4,250,820 bp in length, with a GC content of 43.55% (Table S3). Multiple genes associated with plant growth promotion were identified in the genome, including genes involved in IAA synthesis, phosphate solubilization, siderophore biosynthesis, and root colonization (Table S7). AntiSMASH analysis identified 17 BGCs related to secondary metabolite biosynthesis in the A-5 genome, including non-ribosomal peptide synthetases (NRPS), polyketide synthases (PKS), hybrid NRPS-PKS, and RiPPs, accounting for 15.4% of the total genome. These BGCs encode multiple known antifungal metabolites, including bacillibactin, lichenysin, bacitracin, and fengycin. Additionally, 7 other BGCs showed no similarity to known clusters in the MiBIG database, suggesting the potential for novel bioactive metabolite production (Figure 3B and Figure 5, Table S8). These genomic results were fully consistent with the in vitro functional traits of strain A-5, revealing the genetic basis of its strong biocontrol and plant growth-promoting potential.

### 3.6. Biocontrol Efficacy in Pot Experiment of Strain A-5 Against Watermelon Fusarium Wilt

**Figure 6:**
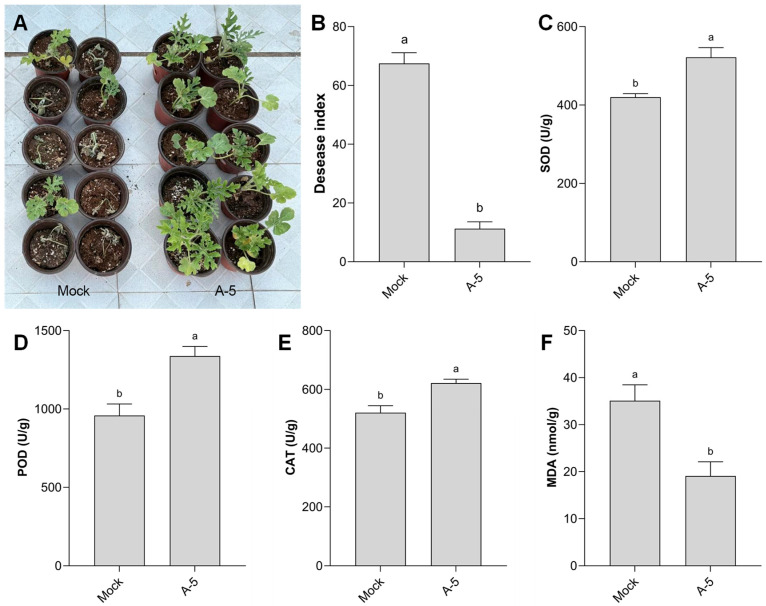
Biocontrol efficacy of *B. sonorensis* A-5 against watermelon Fusarium wilt and its effect on physiological indexes of watermelon seedlings. (**A**) Phenotypic characteristics of watermelon seedlings in the Mock group (*Fon*-infested soil irrigated with sterile water) and A-5 treatment group at 10 days after the final irrigation. (**B**) Disease index of watermelon seedlings under different treatments. (**C**–**F**) Physiological indexes of watermelon leaves in different treatments: (**C**) superoxide dismutase (SOD) activity; (**D**) peroxidase (POD) activity; (**E**) catalase (CAT) activity; (**F**) malondialdehyde (MDA) content. Different letters above bars indicate significant differences (*p* < 0.05).

The greenhouse pot experiment verified the control efficacy of strain A-5 against watermelon Fusarium wilt. The Mock group had a disease incidence of 75.00% and a disease index of 67.50, with severe wilting symptoms on watermelon seedlings. In contrast, the A-5 treatment group showed a dramatic reduction in disease development: the disease incidence dropped to 15.00%, the disease index decreased to 11.25, and the relative control efficacy reached 78.04% (Table 1, Figure 6A,B).

Physiological indicator detection showed that, compared with the Mock group, the activities of SOD, POD and CAT in watermelon leaves of the A-5 treatment group were significantly increased by 32.1%, 41.2% and 14.3%, respectively. Meanwhile, the MDA content in the A-5 treatment group was significantly reduced by 44.7% compared with the Mock group (Figure 6C–F). These results indicated that strain A-5 can enhance the systemic disease resistance of watermelon seedlings by upregulating the activity of defense-related antioxidant enzymes and alleviating oxidative damage to plant cells.

## 4. Discussion

Fusarium wilt of watermelon, caused by *Fusarium oxysporum* f. sp. *niveum* (*Fon*), continues to threaten global watermelon production due to its persistence in soil and the limited effectiveness of chemical fungicides [3,5]. Increasing concerns over fungicide resistance and environmental impact have accelerated the search for eco-friendly biocontrol strategies [15]. Numerous *Bacillus* species (such as *B. subtilis*, *B. amyloliquefaciens*, and *B. velezensis*) have demonstrated strong antagonistic activity against *Fusarium* pathogens [40,41,42,43]. *Bacillus velezensis* SDTB038 was shown to inhibit *Fusarium* crown and root rot of tomato through the production of multiple secondary metabolites, with its fermentation broth achieving a control efficacy of 42.98% [44]. Similarly, *B. amyloliquefaciens* DHA55 effectively suppressed watermelon Fusarium wilt through the production of antifungal lipopeptides, including iturin, surfactin, and fengycin, achieving a biocontrol efficacy of 74.9% in greenhouse trials [16]. However, the biocontrol potential of *Bacillus sonorensis* remains largely unexplored. In this study, we isolated strain A-5 from the watermelon rhizosphere and identified it as *B. sonorensis*. Strain A-5 exhibited strong antagonism against *Fon* both in vitro and in pot experiments, indicating its promise as a novel biocontrol agent.

The direct antifungal activity of strain A-5 is primarily mediated by its secretion of cell wall-degrading enzymes (CWDE), particularly protease and β-1,3-glucanase. β-1,3-glucan and structural proteins constitute the major components of the *Fon* cell wall and are essential for maintaining hyphal morphology, vegetative growth, and pathogenicity [45]. Similar CWDE-mediated antagonism has been reported in *B. velezensis* strains that suppress *Rhizoctonia solani* and *Fusarium* spp. by degrading cell wall polysaccharides and disrupting membrane integrity [46]. Damage to the fungal cell wall and membrane can trigger osmotic imbalance, mitochondrial membrane potential collapse, and excessive reactive oxygen species (ROS) accumulation, ultimately leading to fungal apoptosis [47].

Secondary metabolites also play a central role in A-5-mediated antagonism. Genome mining revealed biosynthetic gene clusters encoding lipopeptides such as fengycin and lichenysin, as well as bacillibactin-type siderophores. Fengycin is known to disrupt fungal membranes and induce ROS-dependent apoptosis [48], while lichenysin exhibits strong biosurfactant and antifungal properties that inhibit spore germination and hyphal growth [49]. Bacillibactin, a catechol-type siderophore, chelates Fe^3+^ with high affinity, limiting iron availability to pathogens and thereby suppressing fungal growth through nutritional competition [50,51]. These BGCs are similar to those identified in other biocontrol *Bacillus* strains. *B. velezensis* SDTB038 was found to contain seven secondary metabolite BGCs, including those for fengycin, surfactin, and bacillibactin, which were linked to its biocontrol activity against tomato Fusarium wilt [44]. Similarly, *B. subtilis* KP3P9 and *B. siamensis* K13C were reported to harbor BGCs for fengycin, bacillibactin, subtilin, and bacilysin, which contributed to their broad-spectrum antifungal activity against multiple *Fusarium* species [52]. Notably, however, the presence of these BGCs in the genome merely reflects biosynthetic potential. Despite the valuable predictions afforded by genome mining, follow-up metabolomic analysis is needed to validate the actual expression and secretion of these metabolites. In addition, volatile organic compounds (VOCs) produced by beneficial microbes, including *Streptomyces* and *Bacillus*, have been shown to inhibit *Fusarium* spp. growth [53,54], consistent with the VOC-mediated inhibition observed in strain A-5.

Beyond direct antagonism, strain A-5 enhanced systemic resistance in watermelon seedlings. Plants treated with A-5 exhibited elevated activities of antioxidant enzymes such as superoxide dismutase (SOD), peroxidase (POD), and catalase (CAT), along with reduced malondialdehyde (MDA) accumulation. These enzymes are central to ROS detoxification and redox homeostasis during pathogen attack [55]. Beneficial microbes such as *Burkholderia vietnamiensis* and *Bacillus* spp. have been shown to induce similar defense responses, enhancing plant resistance to soil-borne pathogens [25,26]. Moreover, A-5 produces indole-3-acetic acid (IAA), which promotes root development and improves plant vigor, further contributing to disease tolerance. This is consistent with findings from multiple studies showing that IAA-producing *Bacillus* strains can enhance plant growth and disease resistance [16,56,57].

When comparing in vitro and in vivo results, strain A-5 exhibited strong in vitro antagonism against *Fon*, with an inhibition rate of 70.15% in dual-culture assays. Under greenhouse conditions, this antagonistic activity translated into a biocontrol efficacy of 78% against Fusarium wilt, as reflected by a significant reduction in disease index compared to the pathogen-only control. This suggests that the strain’s ability to control Fusarium wilt involves more than just direct antifungal activity. The *γ*-PGA-deficient mutant of *B. atrophaeus* NX-12 exhibited enhanced in vitro antifungal activity, its in situ biocontrol efficacy was significantly diminished due to impaired biofilm formation and root colonization, underscoring that direct antagonism alone is insufficient to predict overall biocontrol performance [58]. The final biocontrol outcome attributed the discrepancy to the combined action of multiple mechanisms, including induced systemic resistance, root colonization, and secondary metabolite production [46]. For strain A-5, the higher in vivo efficacy relative to in vitro inhibition may be attributed to additional mechanisms such as the induction of plant defense responses, the production of growth-promoting substances like IAA, and effective rhizosphere colonization facilitated by biofilm formation. These combined effects likely contribute to a more robust suppression of *Fon* infection under complex soil conditions.

Strain A-5 also demonstrated broad-spectrum antifungal activity against *Verticillium dahliae*, *F. fujikuroi*, *Colletotrichum camelliae*, and *Phytophthora citrophthora*, suggesting its potential applicability across diverse crop systems. However, this study has limitations: the specific antifungal compounds responsible for *Fon* suppression have not yet been purified, and the molecular interactions among A-5, *Fon*, and watermelon require further elucidation. Future work should focus on metabolite purification, transcriptomic and metabolomic profiling, and multi-site field trials to validate the practical application of A-5 as a commercial biocontrol agent.

## 5. Conclusions

In this study, we isolated a high-efficiency *Fusarium oxysporum* f. sp. *niveum* (*Fon*)-antagonistic strain A-5 from healthy watermelon rhizosphere, identified as *Bacillus sonorensis* via polyphasic taxonomic analysis. The strain had a 70.15% in vitro inhibition rate against *Fon*, with concentration-dependent antifungal activity of its sterile filtrate, significant *Fon* growth suppression by its volatile compounds, and broad-spectrum antifungal activity. It harbored multiple biocontrol and plant growth-promoting traits, with 17 antimicrobial biosynthetic gene clusters detected in its genome. Pot trials confirmed its 78.04% control efficacy against watermelon Fusarium wilt, as it enhanced watermelon systemic disease resistance by upregulating defense-related antioxidant enzymes. *B. sonorensis* A-5 is a promising biocontrol agent for watermelon Fusarium wilt, providing a novel microbial resource for soil-borne disease management.

## Figures and Tables

**Table 1:** Pot-based control efficacy of different treatments against watermelon wilt.

Treatment	Incidence Rate (%)	Disease Index	Control Effect (%)
Mock	75.00	67.50 a	-
A-5	15.00	11.25 b	78.04

Different letters above bars indicate significant differences (*p* < 0.05).

## Data Availability

The original contributions presented in the study are included in the article material/Supplementary Material. Further inquiries can be directed to the corresponding authors.

## Supplementary Materials

The following supporting information can be downloaded at: https://www.mdpi.com/article/10.3390/jof12040257/s1, Table S1: Basic physicochemical properties of the tested soil for pot experiment; Table S2: Antagonistic activity of candidate bacterial strains against *Fusarium oxysporum* f. sp. *niveum* (*Fon*); Table S3: Collections of *Bacillus* used in this study; Table S4: Inhibitory effect of sterile fermentation filtrate of strain A-5 at different concentrations (0%, 5%, 10%, 20%, 40%) on *Fon* mycelial growth; Table S5: Inhibitory effect of VOCs on *Fon* mycelial growth: inoculated with sterile water, 100 and 200 μL of strain A-5 bacterial suspension, respectively; Table S6: Broad-spectrum antifungal activity of strain A-5 against four phytopathogenic fungi: *Phytophthora citrophthora*, *Verticillium dahliae*, *Fusarium fujikuroi*, and *Colletotrichum camelliae*; Table S7: Plant growth promotion of *Bacillus sonorensis* A-5; Table S8: Biosynthetic gene clusters (BGCs) involved in biosynthesis of secondary metabolites bacterial strain *Bacillus sonorensis* A-5, detected by antiSMASH server.
