# Supplementary material for: Biocontrol Potential and Functional Characteristics of Bacillus sonorensis A-5 Against Watermelon Fusarium Wilt
Source: J Fungi (Basel). 2026 Apr 2;12(4):257. doi: 10.3390/jof12040257 (PMC13117742; doi:10.3390/jof12040257)
Supplement: Supplementary file 1 [file jof-12-00257-s001.zip › jof-4215729-supplementary.pdf]

Supplemantary Materials

Table S1. Basic physicochemical properties of the tested soil for pot experiment.

index	Value
pH value	6. 28 ± 0.09
Total nitrogen (g/kg)	4.21 ± 0.03
Available phosphorus (mg/kg)	15.68 ± 0.85
Available potassium (mg/kg)	117.23 ± 3.46

Table S2. Antagonistic activity of candidate bacterial strains against *Fusarium oxysporum* f. sp. *niveum* (*Fon*).

Strain	Colony diameter (mm)
A-5	25.56 ± 0.85
2-5	40.55 ± 1.42
2-2	34.76 ± 1.27
1-10	39.28 ± 0.69
1-13	41.81 ± 1.11
1-5	50.73 ± 1.04
1-1	49.31 ± 0.49
1-6	48.04 ± 0.96
Mock	85.65 ± 0.37

Table S3. Collections of *Bacillus* used in this study.

Species	Strain ID	Host	Source	Location	Accession numbers	Base Size (bp)	Percent G+C (%)	No. of Proteins	ANI with A-5 (%)	DDH with A-5 (%)
Bacillus sonorensis	A-5	Environment	Soil	China	JBVUNY000000000	4,680,094	43.55	4551	100	100
Bacillus axarquiensis	B-41617	Environment	river	Spain	GCF_001517115	4,091,457	44	3915	78.68	16.9
Bacillus cabrialesii	TE3	Triticum turgidum	seed	Mexico	GCF_004124315	4,064,844	44.1	4039	78.8	16.8
Bacillus halotolerans	ATCC 25096	Environment	soil	Germany	GCF_001517105	4,072,732	44	3902	78.62	16.9
Bacillus inaquosorum	KCTC 13429	NA	NA	South Korea	GCA_000332645	4,342,448	43.5	4431	78.98	17.3
Bacillus malacitensis	NRRL B-41618	Environment	Water	Spain	GCA_001517135	4,186,279	43.5	3957	78.55	16.8
Bacillus mojavensis	KCTC 3706	NA	NA	NA	GCA_000507105	3,935,582	43.7	3944	78.52	16.5
Bacillus rugosus	SPB7	Spongia officinalis	Plant	India	GCF_011745685	4,503,796	43.1	4511	78.66	16.9
Bacillus sonorensis	PMC204	Environment		South Korea	GCF_034055115	4,803,670	45	4751	99.45	91
Bacillus spizizenii	TU-B-10	NA	NA	NA	GCF_000227465	4,205,222	43.8	4297	78.6	17
Bacillus subtilis	ATCC 6051	NA	NA	NA	GCF_000344745	4,215,610	43.5	4345	78.64	16.9

Table S4. Inhibitory effect of sterile fermentation filtrate of strain A-5 at different concentrations (0%, 5%, 10%, 20%, 40%) on *Fon* mycelial growth.

Sterile filtrate concentration	Colony diameter (mm)
5%	81.97 ± 1.32
10%	69.20 ± 1.76
20%	51.80 ± 1.06
40%	17.05 ± 0.26
Mock	90

Table S5. Inhibitory effect of VOCs on *Fon* mycelial growth: inoculated with sterile water, 100 and 200 μL of strain A-5 bacterial suspension, respectively.

Volume of bacterial solution applied	Colony diameter (mm)
100 μL A-5	81.97 ± 1.32
200 μL A-5	69.20 ± 1.76
Mock	75.01 ± 0.34

Table S6. Broad-spectrum antifungal activity of strain A-5 against four phytopathogenic fungi: *Phytophthora citrophthora*, *Verticillium dahliae*, *Fusarium fujikuroi*, and *Colletotrichum camelliae*.

Strain	Colony diameter (mm)
Phytophthora citrophthora (A-5)	19.92 ± 1.03
Phytophthora citrophthora (Mock)	51.18 ± 0.87
Verticillium dahliae (A-5)	34.12 ± 1.27
Phytophthora citrophthora (Mock)	54.14 ± 0.74
Fusarium moniliforme (A-5)	40.08 ± 1.39
Phytophthora citrophthora (Mock)	72.17 ± 0.93
Colletotrichum gloeosporioides (A-5)	40.41 ± 1.29
Phytophthora citrophthora (Mock)	74.48 ± 1.07

Table S7 Plant growth promotion of *Bacillus sonorensis* A-5

Function	Gene	Description	Start	End	Strand	Scaffold
IAA production	trpA_1	Tryptophan synthase alpha chain	192995	193798	-	CJFNHAND_01180
	trpB_1	Tryptophan synthase beta chain	193767	194993	-	CJFNHAND_01181
	trpF	N-(5'-phosphoribosyl) anthranilate isomerase	194974	195654	-	CJFNHAND_01182
	trpC	Indole-3-glycerol phosphate synthase	195623	196387	-	CJFNHAND_01183
	trpD_1	Anthranilate phosphoribosyltransferase	196368	197396	-	CJFNHAND_01184
	trpE	Anthranilate synthase component 1	197368	198915	-	CJFNHAND_01185
	trpD_2	Anthranilate phosphoribosyltransferase	145503	146546	-	CJFNHAND_01479
	trpB_2	Tryptophan synthase beta chain	131539	132729	+	CJFNHAND_01974
	trpA_2	Tryptophan synthase alpha chain	132726	133535	+	CJFNHAND_01975
	trpS	Tryptophan--tRNA ligase	49871	50860	+	CJFNHAND_03065
	trpP	putative tryptophan transport protein	49688	50215	-	CJFNHAND_03193
Phosphate solubilization	phoD_1	Alkaline phosphatase D	113751	115502	+	CJFNHAND_00659
	pstS1	Phosphate-binding protein PstS 1	49122	50039	+	CJFNHAND_03311
	pstA	Phosphate transport system permease protein PstA	51042	51929	+	CJFNHAND_03313
	pstB3_1	Phosphate import ATP-binding protein PstB 3	51949	52770	+	CJFNHAND_03314
	pstB3_2	Phosphate import ATP-binding protein PstB 3	52784	53563	+	CJFNHAND_03315
	phoD_2	Alkaline phosphatase D	62188	63741	-	CJFNHAND_03651
	phoA	Alkaline phosphatase 4	29464	31125	+	CJFNHAND_03949
	phoD_3	Alkaline phosphatase D	33973	35544	+	CJFNHAND_04069
	phoR	Alkaline phosphatase synthesis sensor protein PhoR	54290	56032	-	CJFNHAND_04087
	phoP	Alkaline phosphatase synthesis transcriptional regulatory protein PhoP	56025	56747	-	CJFNHAND_04088

Siderophore production	feuC_1	Iron-uptake system permease protein FeuC	71756	72778	-	CJFNHAND_00069
	feuB_1	Iron-uptake system permease protein FeuB	72771	73775	-	CJFNHAND_00070
	feuA	Iron-uptake system-binding protein	73795	74751	-	CJFNHAND_00071
	fhuD_1	Iron(3+)-hydroxamate-binding protein FhuD	481282	482208	+	CJFNHAND_00459
	feuB_2	Iron-uptake system permease protein FeuB	482355	483305	+	CJFNHAND_00460
	feuB_3	Iron-uptake system permease protein FeuB	59095	60159	+	CJFNHAND_01390
	feuC_2	Iron-uptake system permease protein FeuC	60159	61178	+	CJFNHAND_01391
	feuC_3	Iron-uptake system permease protein FeuC	102028	103044	-	CJFNHAND_01941
	feuC_4	Iron-uptake system permease protein FeuC	103041	104048	-	CJFNHAND_01942
Root colonization	flgG_1	Flagellar basal-body rod protein FlgG	35346	36170	-	CJFNHAND_00038
	flgG_2	Flagellar basal-body rod protein FlgG	36232	37071	-	CJFNHAND_00039
	fliD	Flagellar hook-associated protein 2	1515	2996	+	CJFNHAND_02036
	fliS	Flagellar secretion chaperone FliS	3018	3419	+	CJFNHAND_02037
	fliT	Flagellar protein FliT	3419	3760	+	CJFNHAND_02038
	flhB_1	Flagellar biosynthetic protein FlhB	10802	11080	-	CJFNHAND_02394
	flhF	Flagellar biosynthesis protein FlhF	117790	118905	-	CJFNHAND_02983
	flhA	Flagellar biosynthesis protein FlhA	118902	120938	-	CJFNHAND_02984
	flhB_2	Flagellar biosynthetic protein FlhB	120969	122051	-	CJFNHAND_02985
	fliR	Flagellar biosynthetic protein FliR	122048	122824	-	CJFNHAND_02986
	fliP	Flagellar biosynthetic protein FliP	123117	123782	-	CJFNHAND_02988
	fliM	Flagellar motor switch protein FliM	126410	127120	-	CJFNHAND_02992
	flgG_3	Flagellar basal-body rod protein FlgG	127844	128638	-	CJFNHAND_02994
	fliJ	Flagellar FliJ protein	131135	131578	-	CJFNHAND_02998
	fliG	Flagellar motor switch protein FliG	133644	134660	-	CJFNHAND_03001
	fliF	Flagellar M-ring protein	134674	136275	-	CJFNHAND_03002

	fliE	Flagellar hook-basal body complex protein FliE	136326	136643	-	CJFNHAND_03003
	flgC	Flagellar basal-body rod protein FlgC	136656	137108	-	CJFNHAND_03004
	flgB	Flagellar basal body rod protein FlgB	137108	137497	-	CJFNHAND_03005
	fliW	Flagellar assembly factor FliW	2853	3284	-	CJFNHAND_03764
	flgL	Flagellar hook-associated protein 3	3921	4832	-	CJFNHAND_03766

Table S8 Biosynthetic gene clusters (BGCs) involved in biosynthesis of secondary metabolites bacterial strain *Bacillus sonorensis* A-5, detected by antiSMASH server.

Cluster	Synthetase Type	From	To	Similarity	Metabolite	Function
Cluster 1	NRP-metallophore,NRPS	38,846	90,650	100%	bacillibactin/bacillibactin E/bacillibactin F	Antibacterial, Nutrient uptake, Siderophore during iron deficiency in soil, Microbial competitors
Cluster 2	NRPS	771,416	835,916	100%	lichenysin	Antifungal, Antibacterial, Antibiotic, Induction of ISR
Cluster 3	terpene	1,055,024	1,076,913	-	-	-
Cluster 4	T3PKS	1,123,744	1,164,847	-	-	-
Cluster 5	NRPS	1,299,641	1,366,718	100%	bacitracin	Antibacterial,, Antibiotic
Cluster 6	lassopeptide	1,469,629	1,492,050	-	-	-
Cluster 7	terpene-precursor	1,559,317	1,580,216	-	-	-
Cluster 8	RiPP-like	1,952,544	1,963,905	-	-	-

Cluster 9	NRPS,T1PKS	2,679,045	2,762,666	15%	laterocidine/fengycin	Antifungal, Antibiotic, Induction of ISR
Cluster 10	NRPS	2,830,906	2,873,668	-	-	-
Cluster 11	NI-siderophore	3,063,920	3,097,382	60%	schizokinen	Antifungal, Nutrient uptake, Siderophore during iron deficiency in soil
Cluster 12	azole-containing-RiPP	3,123,898	3,145,832	7%	butirosin A/butirosin B	Antibacterial,, Antibiotic
Cluster 13	terpene-precursor	3,338,033	3,358,923	-	-	-
Cluster 14	betalactone	3,561,330	3,589,837	53%	fengycin	Antifungal, Antibiotic, Induction of ISR
Cluster 15	lanthipeptide-class-ii,cyclic-lactone-autoinducer	3,692,589	3,721,960	100%	amyloliquecidin GF610	Antibacterial, Antibacterial,, Antibiotic
Cluster 16	NRPS	3,900,753	3,955,992	34%	bacitracin	Antibacterial, Antibiotic
Cluster 17	NRPS	4,570,289	4,672,807	34%	fengycin	Antifungal, Antibiotic, Induction of ISR
